# Accessing the impact of harvest weights of *Tenebrio molitor* on amino acid digestibility and metabolizable energy in cecectomized laying hens

**DOI:** 10.1002/jsfa.70196

**Published:** 2025-09-22

**Authors:** Adewunmi Omotoso, Elena Werner, Nils Hautkapp, Markus Rodehutscord, Wolfgang Siegert

**Affiliations:** ^1^ Department of Animal Sciences University of Göttingen Göttingen Germany; ^2^ Alpha‐Protein GmbH Bruchsal Germany; ^3^ Institute of Animal Science University of Hohenheim Stuttgart Germany

**Keywords:** amino acid digestibility, cecectomized laying hen, yellow mealworm (*Tenebrio molitor*), nutrient efficiency, metabolizable energy

## Abstract

**BACKGROUND:**

The effects of harvest weights on composition, amino acid (AA) digestibility and metabolizable energy (ME_N_) of *Tenebrio molitor* (TM) were investigated using cecectomized laying hens. The partially defatted and dried TM variants comprised median individual frass‐free harvest weights of 60, 80, 100, and 120 mg of larvae, and a pupae weight of 125 mg. Diets containing the five TM variants and a basal diet were fed to six cecectomized laying hens over six experimental periods in a 6 × 6 Latin square design.

**RESULTS:**

The AA concentrations relative to crude protein were similar among the TM larvae variants. Crude protein and crude fat contents did not differ between the larvae variants. Pupae had a lower crude protein and a higher crude fat concentration compared to the larvae variants. The AA concentrations in the fat‐free dry matter of TM were low for all harvest weights. Larvae variants did not differ in AA digestibility, whereas pupae showed the highest values. Compared with pupae, larvae had a lower digestibility (*P* ≤ 0.050) for all AA except Arg and Thr in L60; Arg, Asx, Glx, Lys, and Thr in L100, and all AA except Arg, Asx, Glx, Leu, Phe, Pro, and Thr in L120. The ME_N_ did not differ among larvae variants but was higher for the pupae (*P* ≤ 0.050).

**CONCLUSION:**

The present study did not indicate that partially defatted TM larvae of different harvest weights influenced AA digestibility and ME_N_, whereas AA digestibility and ME_N_ of pupae was higher. Feeding partially defatted TM pupae instead of larvae could reduce nitrogenous emissions during egg production, provided that emissions from pupae production remain low. Therefore, optimizing the defatting process for TM pupae may help mitigate challenges associated with the higher fat content, potentially enabling the realization of the benefits from its higher AA digestibility in feed applications. © 2025 The Author(s). *Journal of the Science of Food and Agriculture* published by John Wiley & Sons Ltd on behalf of Society of Chemical Industry.

## INTRODUCTION

Insect‐derived protein sources, such as mealworm larvae (*Tenebrio molitor* L.; TM), are increasingly considered suitable alternatives to established plant‐based sources such as soybean meal.^1^, ^2^ The use of TM for food and feed in the European Union was approved by the European Commission in 2021, allowing dried TM larvae in the market.^3^ Several factors influence the potential of TM as a feed resource. Depending on which feed substrate insects are reared, these factors include the potential for efficient use of arable land and water,^4^, ^5^ and reduced environmental impact compared to the animal production system.^6^, ^7^ Feed ingredients based on TM mainly provide amino acids (AA) and energy expressed as nitrogen‐corrected metabolizable energy (ME_N_) because of the high crude protein (CP) and fat content.^8^

It is generally accepted among nutritionists that the AA requirement of poultry and feed concentrations are best expressed on a digestible AA basis, which requires information on concentrations and digestibility of AA in feed ingredients. The exclusion of post‐ileal fermentation is a prerequisite for determining the AA digestibility in poultry because the post‐ileal AA absorption is negligible, whereas the microbiota in the hindgut alter the AA profile of the digesta.^9^, ^10^ Investigations demonstrated that AA digestibility determination based on the total digestive tract of domestic fowl inaccurately reflects the protein quality of feedstuffs.^11^, ^12^ In domestic fowl, the influence of post‐ileal fermentation can be markedly decreased by cecectomy (i.e. the surgical removal of the ceca) because the ceca are the primary location of microbial fermentation.^13^ After cecectomy, recovered birds do not require additional or different care from non‐cecectomized birds. In the research context, cecectomy allows for accurate digestibility determinations using only a few experimental birds. This is because digestibility can be determined multiple times based on quantified nutrient intake and output over several days.^14^

Variations in the nutritive value, including AA digestibility, of TM‐based feed ingredients have been reported.^15^, ^16^, ^17^, ^18^ Varying nutrient concentrations and AA digestibility on insect‐based meals have been attributed to external factors such as temperature and photoperiodism,^19^, ^20^ feed‐substrate type,^21^, ^22^ methods of AA digestibility determination,^23^ fowl type^24^ and methods of AA digestibility used in different studies.^25^ Developmental stages of insects can also impact nutrient concentrations and AA digestibility. Such effects have been reported in black soldier fly larvae (BSFL).^26^, ^27^ The larval stage is usually investigated when the nutritive value of TM is investigated. Nutrient concentrations of TM differ between larvae and pupae and within the pupae stage.^28^, ^29^ Hence, the AA digestibility of the defatted TM pupae might differ from that of the larvae.

To our knowledge, no previous studies have examined the effects of TM larvae harvest weight on nutrient composition, AA digestibility and ME_N_ in poultry. The present study investigated the nutrient composition of partially defatted TM, which was categorized based on harvest body weight spanning the larvae and pupae phases and the corresponding effect on the AA digestibility and ME_N_ using cecectomized laying hens. The AA digestibility was determined using a regression approach that accounted for undigested AA and specific endogenous AA losses at the same time as excluding basal endogenous AA contributions,^30^ as required for the use for practical feeding purposes.^23^

## MATERIALS AND METHODS

### Ethical statement

The study was performed at the Institute of Animal Science, University of Hohenheim, Germany. All procedures adhered to the German animal welfare regulations and were approved by the Regierungspräsidium Stuttgart under protocol no. V355/19TE.

#### *Tenebrio molitor* meals

**Figure 1:**
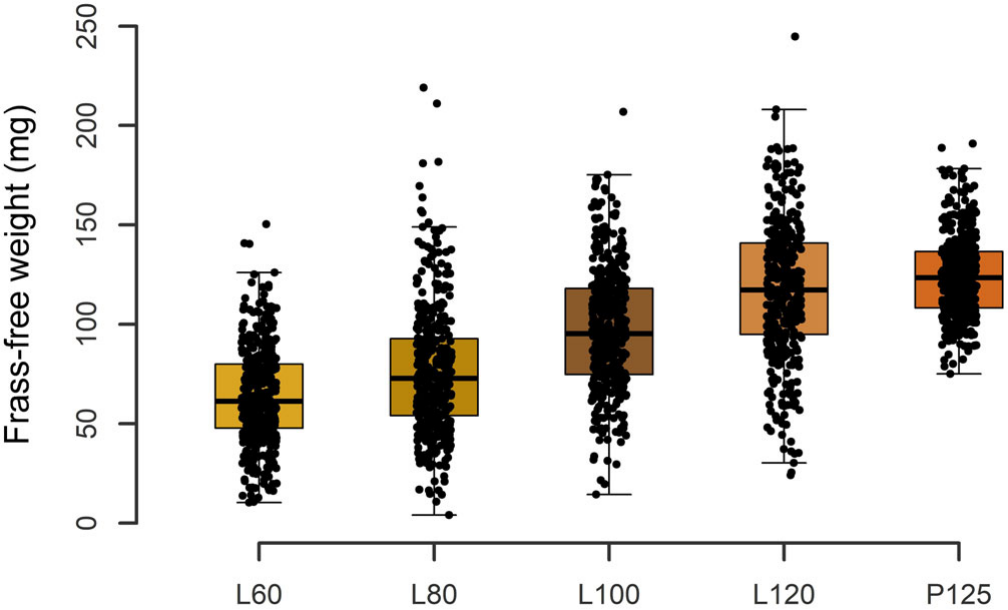
Frass‐free weights of subsamples of 400 individual *Tenebrio molitor* inactivated at target larvae weights of 60 mg (L60), 80 mg (L80), 100 mg (L100) and 120 mg (L120), as well as pupae weights of 125 mg (P125). Dots show the weight of individuals. Boxes represent the interquartile range, horizontal lines within each box indicate the median, and whiskers extend to the furthest data points within 1.5 times the interquartile range.

Larvae of TM were procured from a commercial supplier (Fauna Topics GmbH, Marbach am Neckar, Germany). The TM was reared at Alpha‐Protein GmbH (Bruchsal, Germany) under standardized rearing conditions, which included a temperature range of 23–27 °C and a relative humidity range of 50–60%. The larvae were stocked at 10 individuals per cm.^2^ The TM were weekly supplied with wheat semolina bran and carrot slices for *ad libitum* consumption. The TM were harvested at target average larvae weights of 60 mg (L60), 80 mg (L80), 100 mg (L100) and 120 mg (L120), as well as a pupa weight of 125 mg (P125). The TM weights were confirmed by weekly weighing 400 individuals after removal from the group and a 24‐h feed deprivation. Prior to weighing, larvae were rid of frass (mix of excreta and feed substrate) by sieving with sieves of 1 × 1 mm. The median weight and range of each TM variant were 61.3 mg (10.3–150 mg) for L60, 72.8 mg (4.0–219 mg) for L80, 95.3 mg (14.4–207 mg) for L100, 117 mg (24.1–45 mg) for L120 and 124 mg (75.1–191 mg) for P125 at harvest (Fig. 1). The TM were inactivated by freezing at −20 °C. Subsequently, samples were oven‐dried and partially defatted using a TP 04 mechanical screw press (Maschinenfabrik Reinartz GmbH & Co. KG, Neuss, Germany). This process was conducted at the Internationale Forschungsgemeinschaft Futtermitteltechnik eV (Braunschweig, Germany).

### Experimental diets

The six experimental diets contained either 250 g kg^−1^ cornstarch or 250 g kg^−1^ of one of the five TM variants, which was complemented to a premixture of all ingredients that did not vary among diets (Table 1). The ingredients of the basal diet were formulated to meet or exceed the recommendations of the Gesellschaft für Ernährungsphysiologie^31^ for laying hens with a daily egg production of 60 g and body weight of 1800 g. The main non‐varying ingredients comprised corn, soybean meal, wheat gluten, coarse limestone, and soybean oil. The complete diets were pelleted through a 3‐mm die without steam. The analyzed diet composition (Table 2) confirmed the calculated values.

**Table 1:** Ingredient composition of the basal diet and diets containing the *Tenebrio molitor* variants (g kg^−1^)

Feed ingredients	Basal diet		*Tenebrio molitor* diets
Cornstarch	250		−
TM meal variant (no. 1–5)	−		250
Corn		372.5	
Soybean meal		135	
Wheat gluten		90	
Grass meal		40	
Soybean oil		30	
l‐Lys·sulfate		2	
dl‐Met		0.5	
Premix^a^		20	
Limestone		60	

^a^ Premix provided per kg of diet: 10.2 g of calcium carbonate, 4 g of calcium sodium phosphate, 2.4 g of monocalcium phosphate, 1.4 g of sodium chloride, 0.6 g of sodium carbonate, 10 000 IE of vitamin A (retinyl acetate), 2000 IU of vitamin D3 (cholecalciferol), 20 mg of vitamin E (all‐rac‐α‐tocopheryl acetate), 3 mg of vitamin K3 (menadione sodium bisulfite), 1.6 mg of vitamin B1 (thiamine mononitrate), 4 mg of vitamin B2 (riboflavin), 2.4 mg of vitamin B6 (pyridoxine hydrochloride), 16 μg of vitamin B12 (cyanocobalamin), 20 mg of niacinamide, 6 mg of calcium‐d‐pantothenate, 0.6 mg of folic acid, 0.2 mg of biotin, 250 mg of choline chloride, 60 mg of iron from iron‐(II)‐sulfate monohydrate, 60 mg of manganese from manganese‐(II)‐sulfate monohydrate, 32 mg of zinc from zinc‐oxide, 16 mg zinc from zinc‐sulfate monohydrate, 4 mg of copper from cupric‐(II)‐sulfate pentahydrate, 0.6 mg of iodine from calcium iodate anhydrous, 0.2 mg of selenium from sodium selenite.

**Table 2:** Analyzed compounds in the experimental diets (g kg^−1^ and on a dry matter basis, unless otherwise stated)

Diets	Basal diet	Diets containing *Tenebrio molitor* variants				
Item		L60	L80	L100	L120	P125
Dry matter	927	935	935	937	938	937
Crude protein	184	376	368	373	362	343
Crude fat	59	93	92	86	86	119
Gross energy (MJ kg^−1^)	17.8	19.9	20	19.8	19.5	19.9
Amino acids						
Ala	7.8	22.9	22.4	22.6	22.1	17.3
Arg	9.0	18.9	18.4	18.5	18.3	16.6
Asx	13.1	28.7	27.9	28.3	27.9	26.0
Cys	2.9	4.4	4.3	4.3	4.2	3.9
Glx	46.7	66.0	65.3	66.6	66.6	64.9
Gly	6.9	16.9	16.5	17.0	16.9	14.8
His	4.4	10.4	10.2	10.5	10.5	9.6
Ile	7.3	15.8	15.2	15.6	15.4	14.2
Leu	14.7	28.4	27.8	28.4	28.0	25.9
Lys	7.6	18.1	17.5	17.9	17.3	16.0
Met	3.4	5.7	5.6	5.7	5.6	5.4
Phe	9.2	15.7	15.3	15.7	15.5	14.9
Pro	15.7	27.1	26.3	26.7	26.8	23.1
Ser	8.9	16.9	16.9	17.2	17.2	15.3
Thr	6.0	13.3	13.0	13.2	13.1	11.9
Tyr	5.8	17.0	16.5	17.5	17.8	17.1
Val	7.9	20.1	19.3	19.9	19.7	17.8

*Note*: Asx: Asp + Asn, Glx: Glu + Gln; L60: larvae 60 mg; L80: larvae 80 mg; L100: larvae 100 mg; L120: larvae 120 mg; P125: pupae 125 mg.

### Experimental birds and housing

The experimental birds comprised six cecectomized LSL classic laying hens. Cecectomy was conducted at 20 weeks of age following procedures of Zuber *et al*.^32^ Hens were 54 weeks old at the beginning of the experiment because they had participated previously in not yet published experiments. During the experimental periods, hens were housed individually in metabolism units (89 × 89 × 89 cm) equipped with perches, nesting boxes, water and feeding troughs, wire mesh floors, and walls, allowing them to see their conspecifics. Apart from the experimental periods, the hens were kept together in a floor pen (2.2 × 7.0 m) in the same barn. This pen was equipped with straw and wood shavings as litter, wooden perches, feeding and water troughs, nest boxes, and sand baths. The ambient temperature in the pen was maintained at 20 °C, with lights switched on from 07.00 h to 21.00 h.

### Experimental arrangement and sample collection

The experimental arrangement was a 6 × 6 Latin square, with the six experimental diets being tested in six replicates using six cecectomized hens over six experimental periods ensuring each cecectomized hen received each diet once (see Supporting information, Table S1). This resulted in a total of 36 observations, each with the precision of nutrient balancing based on quantified feed intake and excreta output over several days. This arrangement allowed for considering hen and period effects. Feed intake, eggs and body weight were recorded for each hen. Statistical evaluations of performance data are of limited relevance as a result of the short periods. Each period lasted for 8 days, with an adaptation to the respective diets of 4 days, after which quantitative excreta collection commenced until the end of each period at 07.00 h and 15.00 h daily. The daily feed allowance for the birds was 115 g, which was offered in two meals at 07.00 h and 15.00 h. Feed leftovers and spillage were collected from feeding troughs and water cups, frozen, and later oven‐dried at 105 °C to determine the feed intake on a dry matter (DM) basis. Before collection, excreta were rid of feathers, dander and pellet crumbs, and stored at −20 °C. Prior to freeze‐drying, excreta samples were thawed at 4 °C, weighed and homogenized.

### Chemical analyses

The TM and diet samples were ground through a 1.0‐mm screen for analysis of acid detergent fiber without residual ash (ADF_om_) and the acid detergent insoluble nitrogen (ADF − N) and through a 0.5‐mm screen for the remaining crude nutrients using a centrifugal mill (ZM 200; Retsch GmbH, Haan, Germany). All other analyses in TM and diets were performed after pulverizing using a ball mill (MM 40; Retsch GmbH). Freeze‐dried excreta samples were ground to a fine powder using a vibrating cup mill (Pulverisette 2; Fritsch GmbH, Idar‐Oberstein, Germany).

Nutrient concentrations in the ground diet samples were determined following the descriptions of the Verband Deutscher Landwirtschaftlicher Untersuchungs‐ und Forschungsanstalten^33^ for DM (no. 3.1), crude protein (CP; no. 4.1.1), crude fat (no. 5.1.1b), crude ash (no. 8.1), and ADF_om_ (no. 6.5.2). The Vadopest and Fibretherm analysis systems (C. Gerhardt GmbH & Co. KG, Königswinter, Germany) were used for conducting Kjeldahl digestion and ADF_om_, respectively. The ADF‐N was analyzed as described by Haese *et al*.^34^ The AA analysis was conducted following a modified method described by Siegert *et al*.^35^ The AA analysis procedure involved oxidation in an ice bath using a mixture of hydrogen peroxide, phenolic formic acid solution and phenol. Samples were then hydrolyzed under acidic conditions at 113 °C for 24 h in a mixture of hydrochloric acid and phenol, with norleucine added as a standard. The AA were separated, derivatised and detected using an L‐8900 AA analyzing system (VWR/Hitachi Ltd, Tokyo, Japan). Met and Cys were determined as Met sulfone and cysteic acid, respectively. Gly was not determined in excreta because of potential interference from uric acid hydrolysis. The stationary phase was a Nucleosil 120‐5 C18 column (125 × 4 mm) (VWR, Darmstadt, Germany) with a corresponding guard column. The mobile phase consisted of an 86:14 (v/v) mixture of 0.01 mol L^−1^ sodium acetate buffer (pH 4.5)/methanol at a flow rate of 0.8 mL min^−1^ and a column temperature of 20 °C. During acid hydrolysis of AA, the amide residues of Asn and Gln are cleaved, forming Asp and Glu, respectively.^36^, ^37^ Consequently, it was impossible to differentiate between Asn and Asp as well as Gln and Glu. Hence, these AA are presented as Asx and Glx [i.e. Asx (Asn + Asp) and Glx (Gln + Glu)]. The chitin concentration was calculated using the method described by Marono *et al*.^38^ where $\text{chitin}\;\left(g\;{\mathrm{kg}}^{-1}\right)=\mathrm{ash}\;\text{free}\;\mathrm{ADF}\;\left(g\;{\mathrm{kg}}^{-1}\right)-\left(\mathrm{ADF}-N\times 6.25\right)$. The gross energy was determined using an isoperibolic bomb calorimeter (IKA® C 200; IKA‐Werke GmbH & Co. KG, Staufen, Germany). The calorimeter was calibrated with benzoic acid as the standard. Phosphorus and calcium were analyzed by inductively‐coupled plasma optical spectrometry (Vista Pro; Varian Inc., Macquarie Park, NSW, Australia) at the wavelengths 213.618 for phosphorus and 317.933 for calcium following wet digestion according to Zeller *et al*.^39^ Sulfuric acid and nitric acid were added to the samples and the resulting solutions were heated from 100 to 200 °C for 30 min in a block digestion system equipped with a system to trap nitrous gases (Behr K 20 L; Behr Labor‐Technik GmbH, Germany). After cooling to 100 °C, further nitric acid was added and samples were heated again from 225 to 300 °C for 75 min. The solutions were filtered after cooling to room temperature.

### Calculations and statistical analysis

The AA digestibility of the TM meal variants was determined by initially calculating the AA digestibility of the experimental diet for each observation using:

(1) $$\mathrm{AA}\;\text{digestibility}=\begin{array}{l} {}[\mathrm{AA}\;\text{intake}\left(\mathrm{mg}\;{\mathrm{day}}^{-1}\right)\\ -\;\mathrm{AA}\;\text{excreted}\left(\mathrm{mg}\;{\mathrm{day}}^{-1}\right)/\mathrm{AA}\;\text{intake}\left(\mathrm{mg}\;{\mathrm{day}}^{-1}\right)] \end{array}$$

where the AA intake and AA excreted was calculated as follows:

(2) $$\mathrm{AA}\;\text{intake}\;\left(\mathrm{mg}\;{\mathrm{day}}^{-1}\right)=\mathrm{DM}\;\text{intake}\;\left(g\;{\mathrm{day}}^{-1}\right)\times \mathrm{AA}\;\text{in diet}\;\left(\mathrm{mg}\;g^{-1}\mathrm{DM}\right)$$

(3) $$\mathrm{AA}\;\text{excreted}\left(\mathrm{mg}\;{\mathrm{day}}^{-1}\right)=\begin{array}{l} \mathrm{DM}\;\text{excreted}\left(g\;{\mathrm{day}}^{-1}\right)\\ \times \mathrm{AA}\;\text{in excreta}\left(\mathrm{mg}\;g^{-1}\mathrm{DM}\right) \end{array}$$

The AA digestibility of TM meal variants was determined using a linear regression approach suggested by Rodehutscord *et al*.^30^ This approach estimates the AA digestibility by fitting a linear regression between the amount of AA intake and the amount of AA digested. The slope of the regression line represents the digestibility estimates of the test ingredient, which includes undigested AA and specific endogenous AA, but excludes basal endogenous AA. Hence, digestibility values of the TM meals express the same as the standardized AA digestibility. The model by Siegert *et al*.^40^ was used when the AA digestibility of the TM variants was determined and compared:

(4) $$y_{\mathrm{ijk}}=\mu +\alpha_{i}\times m_{\mathrm{ijk}}+\gamma \times n_{\mathrm{ijk}}+h_{j}+p_{k}+e_{\mathrm{ijk}}$$

where $y_{\mathrm{ijk}}$ is the digested amount of the corresponding AA consumed by the $j_{\mathrm{th}}$ hen during the $k_{\mathrm{th}}$ period fed the meal variant i, μ is the intercept, and α and γ are the variant‐specific and the slope of the digested amount of AA consumed $\left(m_{\mathrm{ijk}}\right)$ and feed intake $\left(n_{\mathrm{ijk}}\right)$ respectively. The model considered the test diet as a fixed effect, where $h_{j}$ and $p_{k}$ were random hen and period effects, respectively, and $e_{\mathrm{ijk}}$ was the error term. Prior to AA digestibility analysis, the AA concentration of the basal diet was subtracted from the AA concentration of diets to equate $m_{\mathrm{ijk}}$ to zero. The statistical analysis was conducted using the MIXED procedure of SAS, version 9.4 (SAS Institute Inc., Cary, NC, USA). Digestible AA concentrations were computed by multiplying AA digestibility estimates with analyzed AA concentrations. Crude fat digestibility and digestible crude fat concentrations were determined accordingly.

The ME_N_ of the TM variants was determined by first calculating the ME_N_ of the experimental diet for each observation using the difference method as performed by Siegert *et al*.^40^ The ME_N_ was calculated as:

(5) $${\mathrm{ME}}_{N}\;\left(\mathrm{MJ}\;{\mathrm{kg}}^{-1}\mathrm{DM}\right)=\left\{\text{gross energy intake}\;(\mathrm{kJ}\;{\mathrm{day}}^{-1}\right)-\;\text{gross energy in excreta}\;\left(\mathrm{kJ}\;{\mathrm{day}}^{-1}\right)-36.5\;\left(\mathrm{kJ}\;{\mathrm{day}}^{-1}\right)\times \left[\left(N\;\text{intake}\;(g\;{\mathrm{day}}^{-1}\right)-\text{excreted}\;N\;(g\;{\mathrm{day}}^{-1}\right)+N\;\text{accretion in eggs}\;\left(g\;{\mathrm{day}}^{-1}\right)]\}/\text{feed intake}\;(\;\left(g\;\mathrm{DM}\;{\mathrm{day}}^{-1}\right)$$

Correction to zero N accretion is considered by the product of N accreted in eggs and 36.5 kJ g^−1^; this represents the analyzed gross energy in the avian urine, as suggested by Titus *et al*.^41^ The N content of the eggs was determined by the product of the total egg mass produced during the collection period and the average N concentration in eggs. An assumed value of 19.2 g kg^−1^ was used for the average N concentration, as suggested by Zuber *et al*.^42^ ME_N_ of cornstarch according to the World's Poultry Science Association (WPSA)^43^ was taken as 16.69 MJ kg. The ME_N_ of the TM variants was calculated as:

(6) $${\mathrm{ME}}_{N}\;\left(\mathrm{MJ}\;{\mathrm{kg}}^{-1}\mathrm{DM}\right)=\frac{a-b+\;c\;\times \;d}{d}$$

where a is the ME_N_ of the diets containing TM variants (MJ kg^−1^ DM), b is the ME_N_ of the basal diet (MJ kg^−1^ DM), c is the ME_N_ of the cornstarch, and d is the proportion of the TM meal in the diets.

## RESULTS

### Chemical composition of *T. molitor* meal variants

The CP concentration was highest in L80 (Table 3) compared to the other variants. The concentration ranges of CP and the first‐limiting AA Met and Cys in the TM variants were 676–760 g kg^−1^ DM, 8.4–9.4 g kg^−1^ DM, and 4.3–6.0 g kg^−1^ DM, respectively. The proportions of Cys and Met in CP were 0.6–0.8 g (16 g N) ^−1^ and 1.2 g (16 g N) ^−1^, respectively. Crude fat and gross energy were highest in P125 (216 g kg^−1^ DM; 25.9 MJ kg^−1^ DM), with small differences among the larvae treatments (95–124 g kg^−1^ DM; 23.6–24.1 MJ kg^−1^ DM). The chitin estimate was highest in L80 variant compared to those in L100, L60, L120 and P125. Phosphorus concentration varied from 11.2 g kg^−1^ DM in L80 to 8.4 g kg^−1^ DM in P125, with the latter showing a 25% lower P concentration than the highest recorded value.

**Table 3:** Analyzed compounds in the *Tenebrio molitor* variants (g kg^−1^ and on a dry matter basis, unless otherwise stated)

Item	*Tenebrio molitor* variants				
	L60	L80	L100	L120	P125
Dry matter	957	955	956	958	955
Crude protein	746	760	755	734	676
Crude fat	124	95	115	107	216
Crude ash	53	54	51	49	41
Chitin estimate	101	108	104	100	59
Gross energy (MJ kg^−1^)	24.1	23.6	24.1	23.9	25.9
Phosphorus	11.1	11.2	10.3	10.0	8.4
Calcium	0.9	0.8	0.8	0.8	0.8
Amino acids					
Ala	58.2	61.0	58.7	56.5	40.9
Arg	36.3	37.7	36.4	36.1	31.3
Asx	59.4	61.3	60.1	59.4	54.1
Cys	5.7	6.0	5.7	5.5	4.3
Glx	79.8	82.0	81.6	83.1	79.5
Gly	38.3	39.8	39.5	39.1	33.0
His	23.5	24.3	24.2	24.2	21.9
Ile	31.1	32.6	32.3	32.0	28.3
Leu	53.0	54.7	53.7	52.8	46.8
Lys	38.7	40.0	39.0	38.3	34.2
Met	9.2	9.4	9.3	9.1	8.4
Phe	25.2	25.8	25.9	25.6	24.0
Pro	45.8	47.0	46.2	45.8	34.4
Ser	33.6	34.4	33.8	33.6	28.7
Thr	28.5	29.4	28.7	28.4	25.1
Tyr	44.1	45.3	46.9	47.5	48.0
Val	44.2	46.4	46.1	45.7	40.0
Amino acids g (16 g N)^−1^					
Ala	7.8	8.0	7.8	7.7	6.1
Arg	4.9	5.0	4.8	4.9	4.6
Asx	8.0	8.1	8.0	8.1	8.0
Cys	0.8	0.8	0.8	0.7	0.6
Glx	10.7	10.8	10.8	11.3	11.8
Gly	5.1	5.2	5.2	5.3	4.9
His	3.2	3.2	3.2	3.3	3.2
Ile	4.2	4.3	4.3	4.4	4.2
Leu	7.1	7.2	7.1	7.2	6.9
Lys	5.2	5.3	5.2	5.2	5.1
Met	1.2	1.2	1.2	1.2	1.2
Phe	3.4	3.4	3.4	3.5	3.6
Pro	6.1	6.2	6.1	6.2	5.1
Ser	4.5	4.5	4.5	4.6	4.2
Thr	3.8	3.9	3.8	3.9	3.7
Tyr	5.9	6.0	6.2	6.5	7.1
Val	5.9	6.1	6.1	6.2	5.9

*Note*: Asx: Asp + Asn, Glx: Glu + Gln; L60: larvae 60 mg; L80: larvae 80 mg; L100: larvae 100 mg; L120: larvae 120 mg; P125: pupae 125 mg.

### Hen performance

The mean feed intake of the hen over the excreta collection phases was 102 g DM day^−1^ (see Supporting information, Table S2) and was not influenced by treatment. The overall mean body weight was 1658 g (see Supporting information, Table S3). The laying rate was on average 97%, with a mean egg mass production of 60.5 g day^−1^ (see Supporting information, Table S4).

### Amino acid digestibility and metabolizable energy of *T. molitor* meal variants

The digestibility of most AA did not differ among larvae variants (*P* ≥ 0.078) (Table 4). P125 exhibited the highest overall AA digestibility, with differences (*P* < 0.050) observed among TM variants for all AA except Met and Cys.

**Table 4:** Amino acid and crude fat digestibility (%) as well as nitrogen‐corrected metabolizable energy (ME_N_; MJ kg^−1 DM^) of the *Tenebrio molitor* variants

	L60	L80	L100	L120	P125	Pooled SEM	Larvae mean
Ala	92.4 b	91.6 b	92.5 b	92.9 b	96.2 a	1.15	92.4
Arg	94.7 a,b	94.2 b	94.9 a,b	95.1 a,b	97.0 a	0.90	94.7
Asx	88.8 b	86.8 b	89.4 a,b	89.2 a,b	93.3 a	1.80	88.6
Cys	87.5	84.9	89.1	87.1	92.9	3.45	87.2
Glx	90.9 b	89.8 b	91.6 a,b	92.2 a,b	94.9 a	1.43	91.1
His	81.8 b	80.6 b	82.6 b	83.6 b	90.1 a	1.63	82.2
Ile	91.8 b	90.9 b	91.8 b	92.0 b	95.4 a	1.15	91.6
Leu	92.8 b	92.2 b	93.2 b	93.5 a,b	96.2 a	1.18	92.9
Lys	91.2 b	90.0 b	91.3 a,b	91.0 b	94.5 a	1.34	90.9
Met	94.8	93.7	95.1	95.7	97.0	1.48	94.8
Phe	92.8 b	91.9 b	93.2 b	93.7 a,b	96.4 a	1.29	92.9
Pro	91.0 b	89.5 b	91.3 b	91.9 a,b	95.7 a	1.43	90.9
Ser	88.5 b	87.5 b	89.1 b	89.7 b	94.8 a	1.80	88.7
Thr	89.4 a,b	88.0 b	90.1 a,b	90.1 a,b	93.9 a	2.01	89.4
Tyr	94.1 b	93.6 b	94.4 b	94.9 b	97.0 a	0.69	94.3
Val	91.0 b	90.1 b	91.1 b	91.6 b	95.5 a	1.11	91.0
Crude fat	96.3	95.5	96.6	97.5	95.5	2.06	96.5
ME_N_	18.9 b	18.7 b	18.9 b	18.1 b	20.9 a	0.72	18.7

*Note*: Different lowercase letters indicate significant differences between variants (*P* ≤ 0.050). Asx: Asp + Asn, Glx: Glu + Gln; L60: larvae 60 mg; L80: larvae 80 mg; L100: larvae 100 mg; L120: larvae 120 mg; P125: pupae 125 mg; Larvae variant mean: mean of the larvae treatments (L60, L80, L100 and L120). Amino acid digestibility values include undigested amino acids from the Tenebrio molitor variants and specific endogenous amino acid losses due to feeding *Tenebrio molitor* variants, but exclude basal endogenous amino acid losses; thus, digestibility values express the same as standardized amino acid digestibility.

Across all AA, the difference in digestibility between P125 and the mean of the larvae treatments was in the range of 2.2%‐units for Met and 7.9%‐units for His. The digestibility of the first‐limiting AA was (P125 and mean of the larvae treatments) 97% and 95% for Met, 93% and 87% for Cys, 95% and 91% for Lys, and 94% and 89% for Thr. Differences in digestibility between P125 and L80 were significant for all AA (*P* ≤ 0.023) except for Met and Cys and between P125 and L60 for all AA (*P* ≤ 0.046) except for Met, Cys, Arg (*P* > 0.051) and Thr (*P* = 0.077). Crude fat digestibility was unaffected by treatment. P125 had the highest ME_N_ concentration (20.9 MJ kg^−1^ DM; *P* = 0.019), whereas values among larvae treatments (average 18.7 MJ kg^−1^ DM) did not differ.

The concentration of digestible AA in most of the larvae variants (Table 5) was numerically higher than in P125. The increases ranged from approximately 3.3 to 16.6 g kg^−1^ DM for all AA, except for Glx, His and Tyr. The concentrations of digestible Glx and His in P125 were within the range observed for larvae, whereas digestible Tyr in P125 was about 1.4–4.0 g kg^−1^ DM higher than in the larvae. The digestible AA concentration of the first‐limiting amino acids (Met, Cys, Lys and Thr) was highest in the L80 and L100 variants, which were similar for all amino acids except Lys, which was 0.7–1.3 g kg^−1^ DM higher in L80 compared to the other larvae treatments.

**Table 5:** Concentrations of digestible amino acid and crude fat of *Tenebrio molitor* variants (g kg^−1^ DM)

	L60	L80	L100	L120	P125
Ala	53.8	55.9	54.3	52.5	39.3
Arg	34.4	35.5	34.6	34.3	30.4
Asx	52.7	53.2	53.7	53.0	50.5
Cys	5.0	5.1	5.1	4.8	4.0
Glx	72.6	73.6	74.8	76.6	75.5
His	19.2	19.6	20.0	20.2	19.7
Ile	28.5	29.6	29.7	29.5	27.0
Leu	49.2	50.4	50.0	49.3	45.0
Lys	35.3	36.0	35.6	34.8	32.3
Met	8.7	8.8	8.8	8.7	8.1
Phe	23.4	23.7	24.1	24.0	23.1
Pro	41.7	42.1	42.2	42.1	32.9
Ser	29.7	30.1	30.1	30.1	27.2
Thr	25.5	25.9	25.9	25.6	23.6
Tyr	41.5	42.4	44.3	45.1	46.5
Val	40.2	41.8	42.0	41.8	38.2
Met+Cys	13.7	13.9	13.9	13.5	12.1
Crude fat	119	91	111	104	206

*Note*: Asx: Asp + Asn, Glx: Glu + Gln; L60: larvae 60 mg; L80: larvae 80 mg; L100: larvae 100 mg; L120: larvae 120 mg; P125: pupae 125 mg.

In the fat‐free DM, the concentration of digestible AA in the larvae variants (Table 6) revealed a similar pattern, with generally higher values in the larvae variants compared to P125 (excluding Glx, His, and Tyr). P125 had the highest concentration of digestible Glx, His, and Tyr. For most other AA, the larvae variants exhibited higher or similar concentrations of digestible AA compared to P125. L80 showed a higher concentration of digestible AA compared to P125 for the majority of AA (Ala, Arg, Cys, Gly, Leu, Pro, Ser, Thr, Val and Met+Cys). Across all larvae variants, concentrations of Ala, Arg, Cys, Gly, Pro, Ser, and Met+Cys were higher than those in P125.

**Table 6:** Nutrient concentrations in *Tenebrio molitor* variants (g kg^−1^ fat‐free dry matter)

	L60	L80	L100	L120	P125
Crude protein	852	840	853	822	862
Crude ash	61	60	58	55	52
Chitin estimate	115	119	118	112	75
Phosphorus	12.7	12.4	11.6	11.2	10.7
Calcium	1.0	0.9	0.9	0.9	1.0
Digestible amino acids					
Ala	66.4	67.4	66.3	63.3	52.2
Arg	41.4	41.7	41.1	40.4	39.9
Asx	67.8	67.7	67.9	66.5	69.0
Cys	6.5	6.6	6.4	6.2	5.5
Glx	91.1	90.6	92.2	93.1	101.4
Gly	43.7	44.0	44.6	43.8	42.1
His	26.8	26.9	27.3	27.1	27.9
Ile	35.5	36.0	36.5	35.8	36.1
Leu	60.5	60.4	60.7	59.1	59.7
Lys	44.2	44.2	44.1	42.9	43.6
Met	10.5	10.4	10.5	10.2	10.7
Phe	28.8	28.5	29.3	28.7	30.6
Pro	52.3	51.9	52.2	51.3	43.9
Ser	38.4	38.0	38.2	37.6	36.6
Thr	32.5	32.5	32.4	31.8	32.0
Tyr	50.3	50.1	53.0	53.2	61.2
Val	50.5	51.3	52.1	51.2	51.0
Met+Cys	17.0	17.0	16.9	16.4	16.2

*Note*: Asx: Asp + Asn, Glx: Glu + Gln; L60: larvae 60 mg; L80: larvae 80 mg; L100: larvae 100 mg; L120: larvae 120 mg; P125: pupae 125 mg.

## DISCUSSION

The findings from the present study revealed no evidence that larvae harvest weights affected the AA profile, AA digestibility or the ME_N_ of partially defatted TM meals. However, pupae showed higher AA digestibility and ME_N_ than larvae. This might be attributed to their higher fat concentration. In the literature, increased dietary oil inclusion (40 g kg^−1^ vs. 20 g kg^−1^) resulted in increased digesta retention time of broiler chickens, which may have contributed to increased digestibility of AA and CP.^44^ It is conceivable that a similar mechanism might have contributed to the higher AA digestibility and ME_N_ in pupae, given their higher fat content compared to larvae.

In general, the determined level of AA digestibility was higher compared to commonly used plant‐based feed ingredients, such as corn, wheat, and soybean meal (e.g. lysine digestibility of 90–95% in the present study, 64–85% for corn,^42^ 69–87% for wheat^2^, ^45^, ^51^, and 85–92% for soybean meal^37^). This higher AA digestibility could potentially contribute to reduced nitrogenous emissions during egg production, provided that dietary AA concentrations are adjusted accordingly. Feeding TM pupae instead of larvae may likewise reduce nitrogenous emissions in laying hens because of their higher digestibility; however, this potential benefit could be offset by the additional emissions associated with producing TM up to the pupal stage. Furthermore, the high crude fat and ME_N_ concentrations of TM pupae may limit their inclusion in commercial diets for laying hens.

Another study on AA digestibility of TM larvae meal determined using the same regression approach as that used in the present study showed lower digestibility estimates with a difference of 5.0% units on average of all reported AA.^15^ The reasons for these deviations are unclear. Possibly, the rearing substrate of the mealworms exerted an influence because another study using the same regression approach showed a considerable impact of the rearing substrate of black soldier fly larvae on AA digestibility.^46^ We are not aware of any study comparing the AA digestibility of TM larvae and pupae. However, a study on the AA digestibility of BSFL and prepupae in laying hens^15^ reported no significant differences for most AA, except for a lower digestibility of prepupae for Ala and Pro and a higher digestibility of the prepupae for Glx.

One other study investigated the effects of developmental stage of insects on the AA digestibility. Do *et al*.^27^ reported that AA digestibility in BSFL numerically tended to increase up to day 23 and then decreased as the larvae approached the pupae stage on day 29. These shifts were significant for some AA. However, Do *et al*.^27^ used a different assay to determine AA digestibility. We only compared treatment effects on digestibility and refrained from comparing digestibility estimates across assays here because methodological details of the assays affect the outcomes.^23^ Hence, the described deviations between impacts of developmental stage of black soldier fly and TM larvae may be explained by assay details in addition to possible differences between the insect species.

In the present study, higher crude fat and ME_N_ concentrations as well as lower concentrations of other chemical constituents in P125 compared to the larvae variants were probably influenced by a less efficient defatting process of the pupae. This process was optimized for larvae. Hence, concentrations of nutrients other than crude fat in the TM variants, including CP and AA concentrations, were not only influenced by development stage, but also by the efficiency of the defatting process. Nonetheless, the data allow for the conclusion that there was no indication of an influence of larvae development stage on CP and AA concentrations in the fat‐free DM (Table 6), as well as the AA profile (Table 3). A discernible shift in P concentration in the fat‐free DM was observed, decreasing progressively as the larvae became heavier, possibly indicating mineral redistribution during metamorphosis.^47^ Yu *et al*.^47^ found that the content of most AA relative to CP across the developmental stages showed an increase in Ala, Asp, Glu, Lys, and Val as the larvae developed into pupae and adults. By contrast, contents of Cys, His, Ile, Leu, Met, Phe, and Trp relative to the CP decreased with advancing ages. Do *et al*.^27^ found that the content of most AA relative to CP was largely unaffected by age of BSFL. However, the content of Ala and Glx relative to CP decreased while the content of Asx, Gly, Tyr and Val relative to CP increased as the larvae became older. This is contradictory to the present study because we found no impacts of TM larvae development stage on the AA profile.

The ME_N_ range for the TM variants in the present study was 18.1–20.9 MJ kg^−1^ DM. This is lower than values reported elsewhere for precision‐fed roosters (21.5–22.1 MJ kg^−1^; no DM reported)^15^ and for broiler chickens (21.6 MJ kg^−1^ DM).^46^ Another study on broiler chickens reported a lower ME_N_ of 16.6 MJ kg^−1^ DM.^16^ Besides rearing substrates of the TM, variations in fat contents of the TM meals under study most likely contributed to that, as suggested by the effect of defatting black soldier fly larvae on ME_N_ in broiler chickens.^48^ The determined ME_N_ in the present study may have been lower than that of non‐cecectomized laying hens because the fermentation of indigestible compounds in the ceca can contribute to the energy supply of laying hens. Such fermentable compounds may include chitin and other prececally indigestible parts of the exoskeleton because the concentrations of short chain fatty acids in the ceca content of laying hens was higher when offered a corn/BSFL‐based diet than a corn/soybean meal‐based diet.^49^ The P125 exhibited a lower chitin concentration compared to the larvae variants, which may have contributed to its higher AA digestibility observed in our study. Albeit, the domestic fowl produces an endogenous acidic chitinase capable of cleaving of chitin, the overall chitin hydrolysis is limited,^50^, ^51^ allowing undigested chitin to pass into the postileal digestive tract. This incomplete enzymatic breakdown may have reduced AA digestibility by encapsulated dietary protein, limiting its accessibility to proteases.^51^ Possibly, the higher digestibility observed in P125 may have been attributable to its lower chitin content and the consequent reduction in protein digestion inhibition. Contributions of protein and fat in the TM meals to possible differences in ME_N_ content between cecectomized and non‐cecectomized laying hens were likely minor because the AA and fat, as the main remaining potentially fermentable constituents in the TM variants, were highly digestible.

Overall, ME_N_ concentrations in common plant‐based feed ingredients used in laying hen diets, such as corn (15.7–17.1 MJ kg^−1^ DM),^41^ wheat (13.0–14.2 MJ kg^−1^ DM)^52^ and soybean meal (8.4–9.9 MJ kg^−1^ DM),^37^ were lower than those of TM meal variants in the present study. The high energy content in partially defatted insect meals may make the formulation of diets for laying hens difficult. Laying hen diets usually contain approximately 11.4 MJ ME_N_ kg^−1^ on as‐fed basis^53^ (equivalent to 13.0 MJ ME_N_ kg^−1^ DM, assuming 880 g DM kg^−1^ DM). Consequently, the high ME_N_ content in the TM meals leads to the necessity to combine TM meals with low‐energy feed ingredients to achieve targeted ME_N_ concentrations, or the inclusion level of TM meals must be restricted.

Prediction equations are necessary for determining ME_N_ concentrations in practical diets because the direct determination is labor‐intensive, time‐consuming, and costly. The equations of the WPSA^43^ are frequently used due to their sufficient accuracy for most purposes and because only the knowledge of CP, crude fat, starch, and sugar content is needed. However, this equation was not developed for use with insect meals. Applying the WPSA equation^43^ to predict the ME_N_ of L60, L80, L100, L120 and P125 resulted in 15.5, 14.7, 15.3, 14.8, and 17.6 MJ kg^−1^ DM, respectively. These values were determined using analyzed CP and crude fat concentrations only because no starch and low sugar content is found in insects. Hence, the equation underestimated the ME_N_ by an average of 3.5 MJ kg^−1^ DM (range 3.3–4.0 MJ kg^−1^ DM), making it barely suitable for accurate feed formulation in its current form for insect meal ME_N_ prediction. Restrictions may also arise when using the prediction equation for diets containing insect meals.

## CONCLUSIONS

The present study gave no evidence that the harvest weight of TM larvae influenced the AA digestibility and ME_N_, but developmental variations in AA digestibility between TM larvae and pupae variants were evident. Feeding partially defatted TM pupae instead of larvae could reduce nitrogenous emissions during egg production, provided emissions from pupae production remain low. Therefore, optimizing the defatting process for TM pupae may help mitigate challenges associated with the higher fat content, potentially enabling the realization of the benefits from its higher AA digestibility in feed applications.

## AUTHOR CONTRIBUTIONS

WS, EW and NH were responsible for study conceptualization. WS was responsible for methodology. WS was responsible for formal analysis. AO and WS were responsible for investigations. WS and MR were responsible for resources. WS was responsible for data curation. AO and WS were responsible for writing the original draft. AO, EW, NH, MR and WS were responsible for reviewing and editing. MR and WS were responsible for supervision. MR and WS were responsible for project administration. WS was responsible for funding acquisition.

## FUNDING

This research received funding from the federal state of Baden‐Württemberg, Germany, *via* the BioPartnerBW program, project number 586081.

## CONFLICTS OF INTEREST

EW and NH are employees of a TM‐producing company. This had no impact on data evaluation and the presentation of results. The other authors declare that they have no conflicts of interest.

## Supporting information


**Table S1.** Experimental arrangement.
**Table S2.** Feed intake during the 4‐day collection phase^†^ (g dry matter day^−1^).
**Table S3.** Hen weight (g).
**Table S4.** Egg production traits.

## Data Availability

The data that support the findings of this study are available from the corresponding author upon reasonable request.
