# Supplementary material for: Accessing the impact of harvest weights of Tenebrio molitor on amino acid digestibility and metabolizable energy in cecectomized laying hens
Source: J Sci Food Agric. 2025 Sep 22;106(1):665–74. doi: 10.1002/jsfa.70196 (PMC12623279; doi:10.1002/jsfa.70196)
Supplement: Supplementary file 1 — Table S1. Experimental arrangement. Table S2. Feed intake during the 4‐day collection phase† (g dry matter day−1). Table S3. Hen weight (g). Table S4. Egg production traits. [file JSFA-106-665-s001.pdf]

Accessing the impact of harvest weights of *Tenebrio molitor* on amino acid digestibility and metabolizable energy in cecectomized laying hens

Supplemental file

Table of contents

Supplementary Table 1. Experimental arrangement 2

Supplementary Table 2. Feed intake 3

Supplementary Table 3. Hen weight 4

Supplementary Table 4. Egg production traits 5

Supplementary Table 1. Experimental arrangement

		Hen					
		1	2	3	4	5	6
Period	1	L60	L80	L100	L120	P125	BD
	2	BD	L60	L80	L100	L120	P125
	3	P125	BD	L60	L80	L100	L120
	4	L120	P125	BD	L60	L80	L100
	5	L100	L120	P125	BD	L60	L80
	6	L80	L100	L120	P125	BD	L60

L60: Diets containing *Tenebrio molitor* variant larvae 60 mg; L80: Diets containing *Tenebrio molitor* variant larvae 80 mg; L100: Diets containing *Tenebrio molitor* variant larvae 100 mg; L120: Diets containing *Tenebrio molitor* variant larvae 120 mg; P125: Diets containing *Tenebrio molitor* variant pupae 125 mg; BD: basal diet

Supplementary Table 2. Feed intake during the 4-day collection phase[†] (g dry matter d^{-1})

Period	Hen	Feed intake	Period	Hen	Feed intake
1	1	100	4	1	106
	2	103		2	105
	3	101		3	106
	4	101		4	104
	5	98		5	102
	6	103		6	106
2	1	90	5	1	96
	2	105		2	107
	3	107		3	107
	4	106		4	103
	5	104		5	103
	6	101		6	106
3	1	59	6	1	97
	2	104		2	106
	3	107		3	107
	4	102		4	101
	5	103		5	104
	6	105		6	107

[†]*P* values for main effects: Diet $P = 0.178$; Hen $P = 0.014$; Period $P = 0.348$.

Supplementary Table 3. Hen weight (g)

Period		Arithmetic mean	Minimum	Maximum	SEM
1	B	1692	1543	1863	45
	C	1768	1633	1888	39
	E	1759	1603	1923	50
2	B	1640	1474	1748	43
	C	1696	1498	1770	44
	E	1689	1498	1784	45
3	B	1694	1580	1762	28
	C	1673	1495	1754	41
	E	1660	1456	1733	43
4	B	1619	1430	1717	43
	C	1633	1470	1718	36
	E	1626	1460	1698	37
5	B	1580	1420	1683	46
	C	1605	1448	1695	40
	E	1599	1458	1697	41
6	B	1627	1452	1741	46
	C	1645	1503	1754	38
	E	1639	1459	1761	51

B: Begin of the period; C: Begin of excreta collection; E: End of the period

Supplementary Table 4. Egg production traits

Period	Hen	Eggs laid per 8 days	Mean egg weight† (g)	Daily egg mass‡ (g d^{-1})
1	1	8	63.9	63.9
	2	8	64.0	64.0
	3	7	64.3	56.2
	4	8	60.6	60.6
	5	8	62.8	62.8
	6	7	61.3	53.7
2	1	8	63.8	63.8
	2	8	63.7	63.7
	3	8	66.8	66.8
	4	8	64.1	64.1
	5	8	60.0	60.0
	6	8	62.5	62.5
3	1	6	62.0	46.5
	2	8	60.2	60.2
	3	8	65.5	65.5
	4	8	61.5	61.5
	5	8	60.1	60.1
	6	8	61.7	61.7
4	1	8	63.7	63.7
	2	8	61.9	61.9
	3	8	63.0	63.0
	4	8	60.5	60.5
	5	8	59.4	59.4
	6	8	60.6	60.6
5	1	7	62.6	47.0
	2	8	62.3	62.3
	3	8	62.7	62.7
	4	8	61.3	61.3
	5	7	62.1	54.3
	6	8	59.6	59.6
6	1	8	62.1	62.1
	2	8	64.9	64.9
	3	7	61.1	53.5
	4	8	62.9	62.9
	5	8	60.2	60.2
	6	8	60.9	60.9

†*P* values for main effects: Diet $P = 0.485$; Hen $P = 0.005$; Period $P = 0.156$.
‡*P* values for main effects: Diet $P = 0.863$; Hen $P = 0.540$; Period $P = 0.469$.
